# Dataset for estimation of muscle Dysmorphia in individuals from Colombia

**DOI:** 10.1016/j.dib.2020.105967

**Published:** 2020-07-03

**Authors:** Isaac Kuzmar, José Consuegra, Jezael Jiménez, Einer López, José Hernández, Ana Noreña-Peña

**Affiliations:** a Simón Bolívar University of Colombia, Cl. 58 #55-132, Barranquilla, Atlántico, Colombia; b University of Alicante, s/n, 03690 San Vicente del Raspeig, Alicante, Spain

**Keywords:** Body dysmorphic disorder, Food supplement, Obesity, Overweight

## Abstract

This paper presents data collected through a questionnaire for the estimation of body dysmorphic disorder known as vigorexy, weight level and levels of exercise dependence in individuals from Barranquilla, Colombia who attend local gyms, based on their physical activity, food supplements eating habits, psychological pressure, and risk of muscle dysmorphia. The Data contains 8 tributes and 200 records; labelling obesity range according to WHO into normal, overweight or obesity. All data was collected in person and directly from users. This data can be used to generate scientific research and intelligent computational tools to identify the obesity level and muscle dysmorphia risk of an individual and to build recommender systems that monitor health and mental status.

Specifications Table

**Table:** 

Subject	Medicine
	Nutrition
Specific subject area	Biology
Type of data	Text
	Table
	Figure
How data were acquired	Survey. *The questionnaire is provided as a supplementary file*
Data format	Raw
	Filtered
Parameters for data collection	Data was retrieved from physical in person survey and processed including missing and atypical data deletion, and data normalization
Description of data collection	Labelling process was performed based on WHO and a binary logistic regression model to predict our outcome (high risk of Muscle Dysmorphia symptoms
Data source location	City/Town/Region: Barranquilla
	Country: Colombia
	Latitude and longitude (and GPS coordinates) for collected samples/data:] 10°58′6.74″ N −74°46′52.75″ W
Data accessibility	Kuzmar, Isaac (2020): Dataset for estimation of muscle dysmorphia in individuals from Colombia. figshare. Dataset. https://doi.org/10.6084/m9.figshare.12482516.v2

Value of the Data

• This data presents information from Barranquilla, Colombia that can be used for the estimation of obesity levels, muscle dysmorphia and levels of exercise dependence in individuals.
• The data can be used for estimation using six categories, allowing a detailed analysis of the affectation level of an individual.
• The data can validate the impact of several factors that propitiate the apparition of weight and muscle dysmorphia known as vigorexy problems.

## 1 Data description

An obsessive mental body dysmorphic disorder known of a subtype of muscle dysmorphia is related with eating disorders; this muscle dysmorphia sometimes is called "vigorexy", "bigorexia", "megarexia", or "reverse anorexia", and consists of the delusional or exaggerated belief that one's own body is too small, too skinny, insufficiently muscular, or insufficiently lean, although in most cases, the individual's build is normal or even exceptionally large and muscular already [1,2,3].

**Fig. 1:**
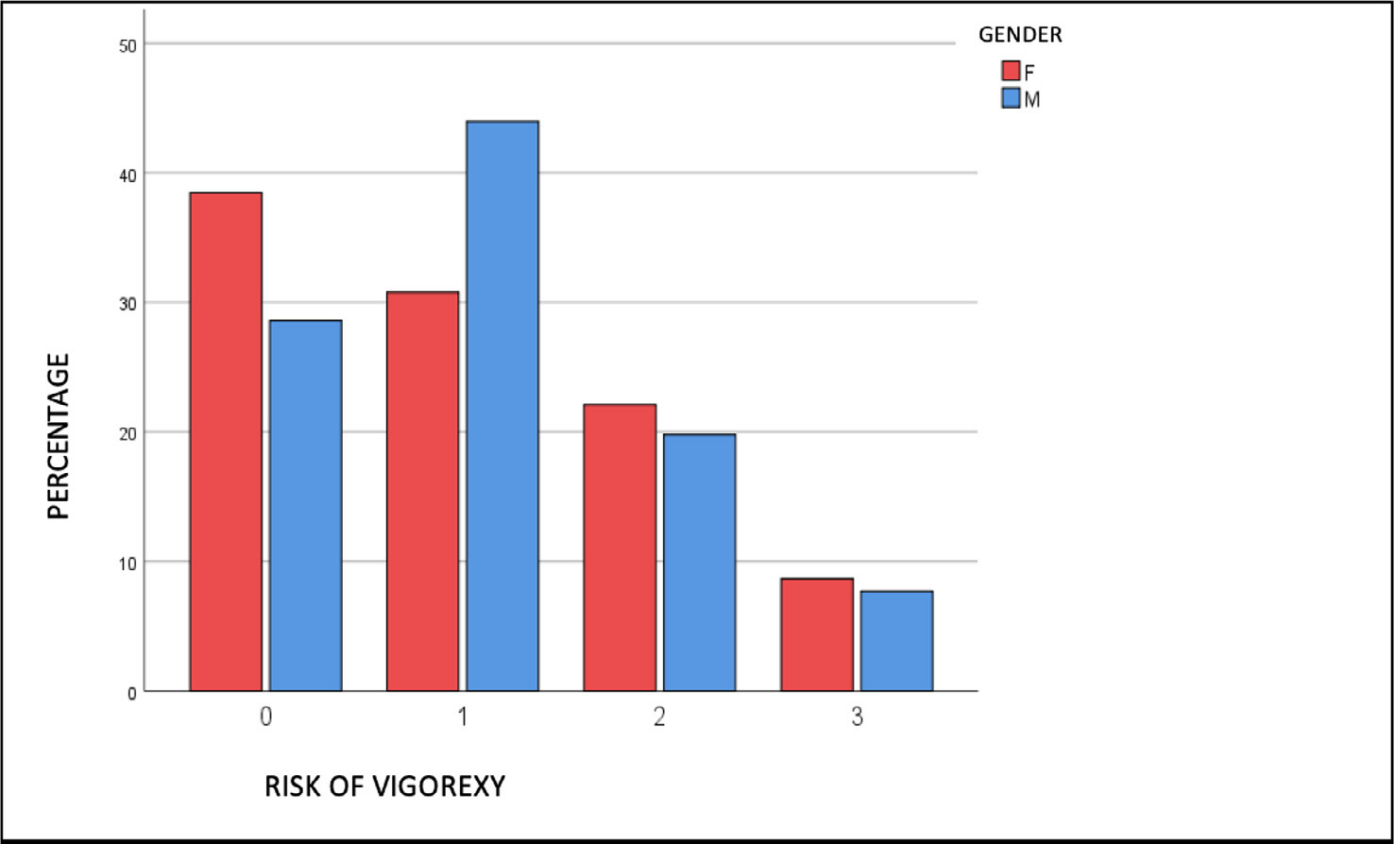
Risk of muscular – body dysmorphic disorder by gender.

**Fig. 2:**
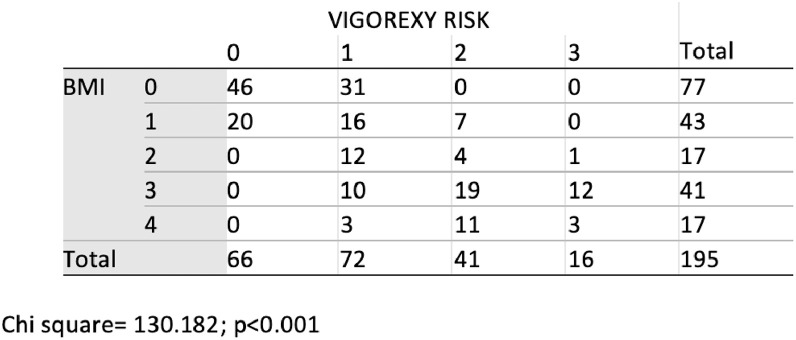
Scrutinizing cross table BMI* risk of muscular dysmorphia. Raw data of Fig. 2 is available at: Kuzmar, Isaac (2020), “Data for: Dataset for estimation of muscle dysmorphia in individuals from Colombia”, Mendeley Data, v1 http://dx.doi.org/10.17632/8652jgm7p5.1 and/or I. Kuzmar, Dataset for estimation of muscle dysmorphia in individuals from Colombia, (2020). https://doi.org/10.6084/m9.figshare.12482516.v2 and/or I. Kuzmar, Dataset for estimation of muscle dysmorphia in individuals from Colombia, (2020). https://doi.org/10.6084/m9.figshare.12482516.v2.

This paper contains data for the estimation of muscle dysmorphia, weight level and levels of exercise dependence in individuals from Barranquilla, Colombia, with ages between 20 and 49 years and diverse eating habits and muscle dysmorphia condition as determined by [4,5], data was collected using a survey (see Table 1) where data collectors filled the document with each participant, then the information was processed obtaining 8 attributes and 200 records, after a data process described in Fig. 1, Fig. 2. The attributes related with eating habits are: gender (G), age (A), Weight(W), Height (H), BMI, food supplements consumption (FSC), physiological condition (PC), and risk of muscle dysmorphia (RMD). The data contains quantitative and qualitative data, so it can be used for analysis based on algorithms of classification, prediction, segmentation and association. Data is available in SAV format to be used with SPPS tool. The questionnaire in English language is provided as a supplementary file. All the raw data related to survey and raw data for each graph, chart is available at: Kuzmar, Isaac (2020), “Data for: Dataset for estimation of muscle dysmorphia in individuals from Colombia”, Mendeley Data, v1 http://dx.doi.org/10.17632/8652jgm7p5.1 and/or I. Kuzmar, Dataset for estimation of muscle dysmorphia in individuals from Colombia, (2020). https://doi.org/10.6084/m9.figshare.12482516.v2

**Table 1:** Questionnaire.

Questions	Possible answers
What is your gender?	1. MALE
	2. FEMALE
	3. PREFER NOT TO ANSWER
What is your age?	1. < 20
	2. 20–29
	3. 30–34
	4. 35–44
	5. > 45
Height	Value in metre
BMI	0. 20–22.49
	1. 22.50–24.99
	2. 25–27.49
	3. 27.50–29.99
	4. > 30
Do you consume food supplements?	1. YES
	2. NO
Have you felt guilt about your lack of adherence to the diet?	1. YES
	2. NO
Risk of muscle dysmorphia	0 = LOW RISK
	1 = MEDIUM RISK
	2 = HIGH RISK
	3 = VERY HIGH RISK

## 2 Experimental design, materials, and methods

The recollection of information was made directly through an in-person filled survey where users had evaluated their physical activity, eating habits and some aspects that helped to identify their muscle dysmorphia risk. The survey was collected over 12 weeks. Inclusion criteria: People who do physical activity, People who consume food supplements, Persons of legal age. Exclusion criteria: people who are not physically active, people who do not consume food supplements, BMI<20. In Table 1, the questions of the survey are presented.

After all data was collected, then data was pre-processed, so it could be used for different techniques of data processing. *N* = 200 records were collected and the data was labelled using equation (4).

Overweight and obesity [6], have been a problem in Colombia [7]. The Medium Risk of Muscle Dysmorphia (Vigorexy) [8,9,10] is higher in Male (>40%) than Female (+30%) gender (see Fig. 1). Doing a simple and initial analysis of the data using the SPSS program obtained through the survey, we appreciate that there is a 9,7% of High Risk of Muscle Dysmorphia [5,6,7] with a BMI = 25–27.59 and a 6.2% of Very High Risk of Vigorexy [5,6,7] with a BMI = 27.50–29.99 (Chi square = 130.182, *p* < 0.001) (see Fig. 2). Raw data available at: Kuzmar, Isaac (2020), “Data for: Dataset for estimation of muscle dysmorphia in individuals from Colombia”, Mendeley Data, v1 http://dx.doi.org/10.17632/8652jgm7p5.1 and/or I. Kuzmar, Dataset for estimation of muscle dysmorphia in individuals from Colombia, (2020). https://doi.org/10.6084/m9.figshare.12482516.v2

## Declaration of Competing Interest

The authors declare that they have no known competing financial interests or personal relationships which have, or could be perceived to have, influenced the work reported in this article.
